# Quantitative optimization of drainage strategy of coalbed methane well based on the dynamic behavior of coal reservoir permeability

**DOI:** 10.1038/s41598-020-77148-1

**Published:** 2020-11-20

**Authors:** Xinlu Yan, Songhang Zhang, Shuheng Tang, Zhongcheng Li, Qian Zhang, Jingyu Wang, Zhiyu Deng

**Affiliations:** 1 School of Energy Resources, China University of Geosciences, Beijing, 100083 China; 2 MOE Key Lab of Marine Reservoir Evolution and Hydrocarbon Accumulation Mechanism, China University of Geoscience, Beijing, 100083 China; 3 Beijing Key Laboratory of Unconventional Natural Gas Geological Evaluation and Development Engineering, Beijing, 100083 China; 4 China United Coalbed Methane Corporation Ltd, Beijing, 100011 China

**Keywords:** Energy infrastructure, Energy science and technology, Energy harvesting, Energy infrastructure, Fossil fuels

## Abstract

The development of coalbed methane (CBM) is not only affected by geological factors, but also by engineering factors, such as artificial fracturing and drainage strategies. In order to optimize drainage strategies for wells in unique geological conditions, the characteristics of different stages of CBM production are accurately described based on the dynamic behavior of the pressure drop funnel and coal reservoir permeability. Effective depressurization is achieved by extending the pressure propagation radius and gas desorption radius to the well-controlled boundary, in the single-phase water flow stage and the gas–water flow stage, respectively, with inter-well pressure interference accomplished in the single-phase gas flow stage. A mathematic model was developed to quantitatively optimize drainage strategies for each stage, with the maximum bottom hole flow pressure (BHFP) drop rate and the maximum daily gas production calculated to guide the optimization of CBM production. Finally, six wells from the Shizhuangnan Block in the southern Qinshui Basin of China were used as a case study to verify the practical applicability of the model. Calculation results clearly indicate the differences in production characteristics as a result of different drainage strategies. Overall, if the applied drainage strategies do not achieve optimal drainage results, the coal reservoir could be irreversibly damaged, which is not conducive to expansion of the pressure drop funnel. Therefore, this optimization model provides valuable guidance for rational CBM drainage strategy development and efficient CBM production.

## Introduction

Gas is stored in micropores on the coal surface in coalbed methane (CBM) reservoirs, mainly via adsorption^1,2^. In order to release CBM from the coal and produce gas through the cleat system, the bottom hole flow pressure (BHFP) is commonly reduced by dewatering, to reduce the reservoir pressure to below the critical desorption pressure. Therefore, under-saturated coal reservoirs can be divided into the drainage area and the desorption area based on the critical desorption pressure^3,4^.

Drainage strategies have a large impact on CBM production. A low BHFP drop rate results in an extended gas production cycle, with uneconomical extraction times typically required^5^. In contrast, a high BHFP drop rate can cause problems such as stress-sensitivity, coal-fines migration and gas–water flow in the early stage of production, which are not beneficial for efficient CBM production^6–12^. Firstly, in the single-phase water flow stage, the effective stress on the coal reservoirs increases with the rapid extraction of water in the coal seam, and pores and cleats are compacted. As a result, fractures are closed and reservoir permeability is reduced. Secondly, coal is easily broken into pieces by continuous washing and the generation and migration of coal fines block the cleat system flow channel, eventually causing irreversible damage to the reservoir. Thirdly, in the early stage of CBM production, the rapid drop in BHFP causes the reservoir pressure to be lower than the critical desorption pressure, causing CBM to desorb and migrate into the cleats. The flow state in coal reservoirs includes both gas–water flow near the well and single-phase water flow in the far well zone and as a result, the flow resistance greatly increases due to the gas–water interfacial force. More importantly, the desorbed bubbles or water also block the flow channel, resulting in a gas or water lock effect. All of these conditions slow the fluid flow, causing inefficient CBM production. Finally, most of the pressure-drop occurs in the two-phase region, while the effect of the pressure-gradient in the single-phase region is minimal^6^. Therefore, optimization of the drainage strategy is necessary to achieve efficient high-yield CBM production.

Currently, the available studies on optimizing drainage strategies mainly concentrate on three aspects: (1) The use of numerical simulation software, such as COMET3, SIMDWIN, and ECLIPSE, in combination with geological data and drainage data, to establish optimal reservoir physical parameters by historical matching. Then, the sensitivity of the drainage rate to production characteristics can be analyzed, allowing the most reasonable drainage strategy under specific geological conditions to be optimized and applied^13,14^. (2) Based on theoretical analysis methods such as seepage theory, material balance equations and rock mechanics, mathematical models have been constructed, with the drainage strategies for different production stages obtained using numerical methods^15–17^. (3) According to the production characteristics of the CBM wells in the area of interest, typical parameters have been extracted from complex drainage curves. By analyzing the coupling relationship between typical parameters, geological factors and engineering factors, the main factors affecting production can be analyzed, allowing reasonable drainage strategies to be developed^18,19^. However, these optimal strategies are essentially qualitative or semi-quantitative. Furthermore, there have been few discussions on the coupling relationship between pressure drop funnel expansion characteristics and the mechanism of permeability dynamic behavior on productivity, despite the importance of these factors for accurately guiding CBM production.

Therefore, this study focuses on optimizing drainage strategies by considering the law of pressure propagation and the dynamic behavior of permeability in different production stages. A principle for BHFP and gas production control is proposed and a mathematical model for the optimization of drainage strategies is established, which are then applied to CBM wells in the Shizhuangnan Block (China) to verify the applicability of this method.

## Model assumptions

### CBM production stages

**Figure 1:**
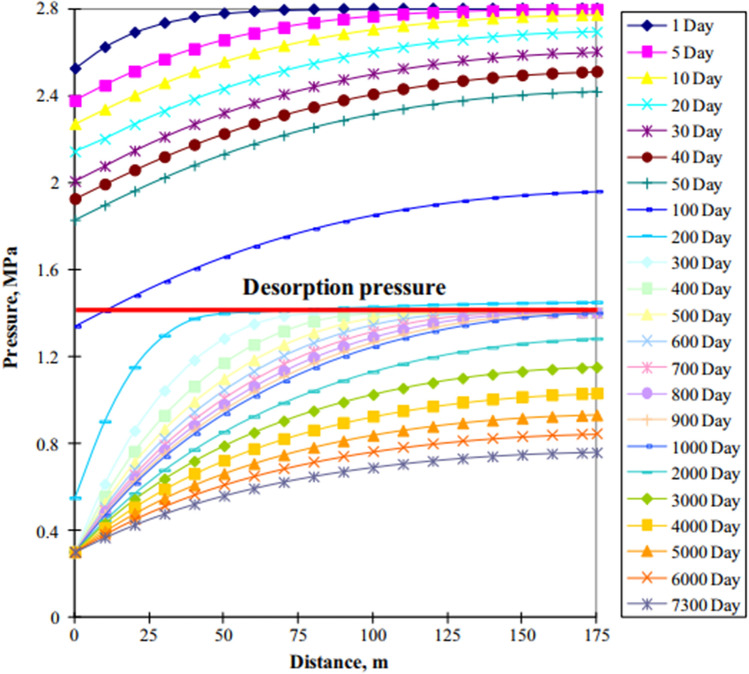
Characteristics of pressure drop funnel for CBM well by numerical simulation^5^.

The optimization of drainage strategies requires the division of production stages into the single-phase water flow stage, gas–water flow stage, and single-phase gas flow stage^8^. Xu et al.^5^ used numerical simulation and analysis methods to characterize the dynamic changes in pressure drop funnels during CBM development^5^ (Fig. 1). Therefore, the characterization of a reasonable pressure drop funnel in different production stages has been described in detail.

**Figure 2:**
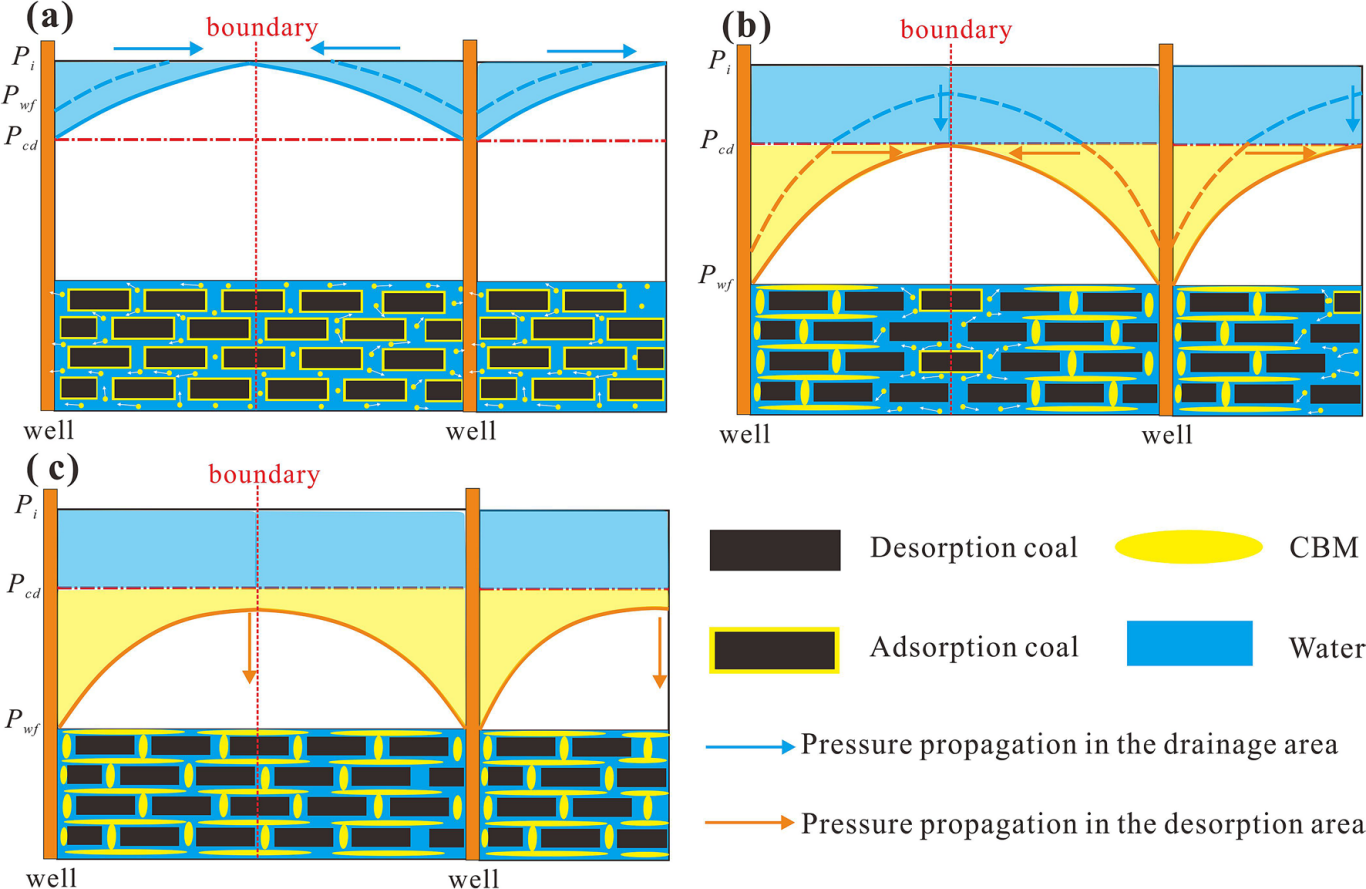
Pressure drop funnel sketch map and the classification of drainage stages with pressure propagation shown (**a**) in the single-phase water flow stage, (**b**) in the gas–water flow stage, (**c**) in the single-phase gas flow stage.

**Figure 3:**
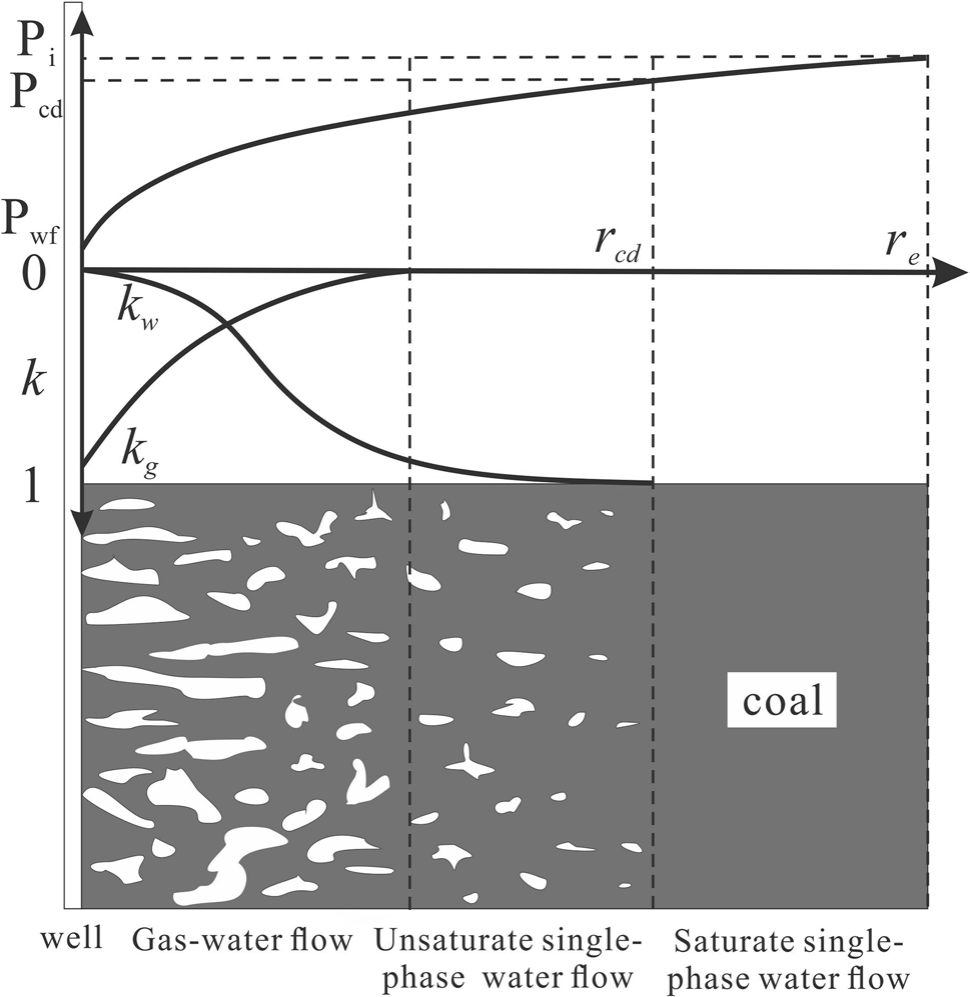
Illustration of pressure propagation and relative permeability in the gas–water flow stage. $k_{w}$ and $k_{g}$ refer to water and gas relative permeability, respectively.

When the reservoir pressure is greater than the critical desorption pressure, CBM cannot desorb and the pores fill with water. Thus, in the single-phase water flow stage, the focus of drainage is to maximize the pressure propagation radius so that the pressure propagation radius reaches the well-controlled boundary ($r_{e}$) (Fig. 2a). The well-controlled boundary generally refers to half of the well spacing due to the well-pattern development mode. Once this occurs, BHFP should drop below the critical desorption pressure and the adsorbed gas then releases into the cleat system. At which point, gas and water coexist in the pores, forming two distinct regions in the reservoir, the gas–water two-phase region and the single-phase water region (Fig. 3). CBM gradually dominates the pores with continued production and therefore, the key in this stage is to gradually expand the desorption radius ($r_{cd}$) until $r_{e}$ is reached (Fig. 2b). Ultimately, the multi-well pressure interference accelerates depressurization and methane desorption, which is conducive to enhanced recovery and CBM production enters the single-phase gas flow stage (Fig. 2c).

### Basic assumptions

CBM development is affected by many factors. In the case of low permeability coal reservoirs, artificial measures like hydrofracturing are required to increase reservoir permeability and to depressurize sufficiently, with artificial fractures produced along the direction of major principal stress. Although artificial fractures are beneficial to fluid flow in the drainage area, the limited fracturing range cannot increase the permeability of the whole coal seam. Moreover, coal fine migration is a common phenomenon that blocks the flow channel during the CBM production process. Therefore, skin factor is taken into account representing the comprehensive impact on reservoir permeability, which can be accurately measured by well testing. In addition, the established mathematical model is based on the some basic assumptions: (a) the coal seam is homogeneous and uniformly thick; (b) gas in the coal seam obeys the gas state equation and the Langmuir equation; (c) fluid flow conforms to Darcy’s law; (d) CBM well production is not affected by external water; (e) dynamic porosity is considered to be a self-regulating coal seam effect^20^:

1 $$\left\{ {\begin{array}{*{20}l} {\varphi_{1} = \varphi_{i} \left[ {1 - C_{f} \left( {P_{i} - P_{w} } \right)} \right]} \hfill & {\quad P \ge P_{cd} } \hfill \\ {\varphi_{2} = \varphi_{i} \left[ {1 - C_{f} \left( {P_{cd} - P_{g} } \right) + \varepsilon_{max} \left( {\frac{{P_{cd} }}{{P_{L} + P_{cd} }} - \frac{{P_{g} }}{{P_{L} + P_{g} }}} \right)} \right]} \hfill & {\quad P < P_{cd} } \hfill \\ \end{array} } \right.$$

where $\varphi_{i}$ is the initial porosity of coal reservoir, dimensionless; $\varphi_{1}$ and $\varphi_{2}$ are the dynamic porosity in the drainage area and desorption area, respectively; $C_{f}$ is the cleat-volume compressibility, MPa^-1^; $\varepsilon_{max}$ is the maximum volumetric strain, dimensionless;$P_{i}$ is the initial reservoir pressure, MPa;$P_{cd}$ is the critical desorption pressure, MPa; $P_{w}$ is the pressure profile in the drainage area, MPa; $P_{g}$ is the pressure profile in the desorption area, MPa.

### Dynamic behavior of reservoir permeability

Reservoir permeability is dynamic. During CBM development, the coal reservoir is damaged by the effective stress effect, with recovery occurring due to the matrix shrinkage effect and the slippage effect. Effective stress induced permeability can be mathematically expressed as shown in Eq. (2), where permeability decreases with the increase in effective stress^21^:

2 $$k_{eff} = k_{i} e^{{ - C_{f} \left( {\frac{1 + v}{{1 - v}}} \right)\left( {P_{i} - P_{w} } \right)}}$$

where $k_{eff}$ is the dynamic permeability induced by the effective stress effect, mD; $k_{i}$ is the initial reservoir permeability, mD; $v$ is Poisson’s ratio, dimensionless.

**Figure 4:**
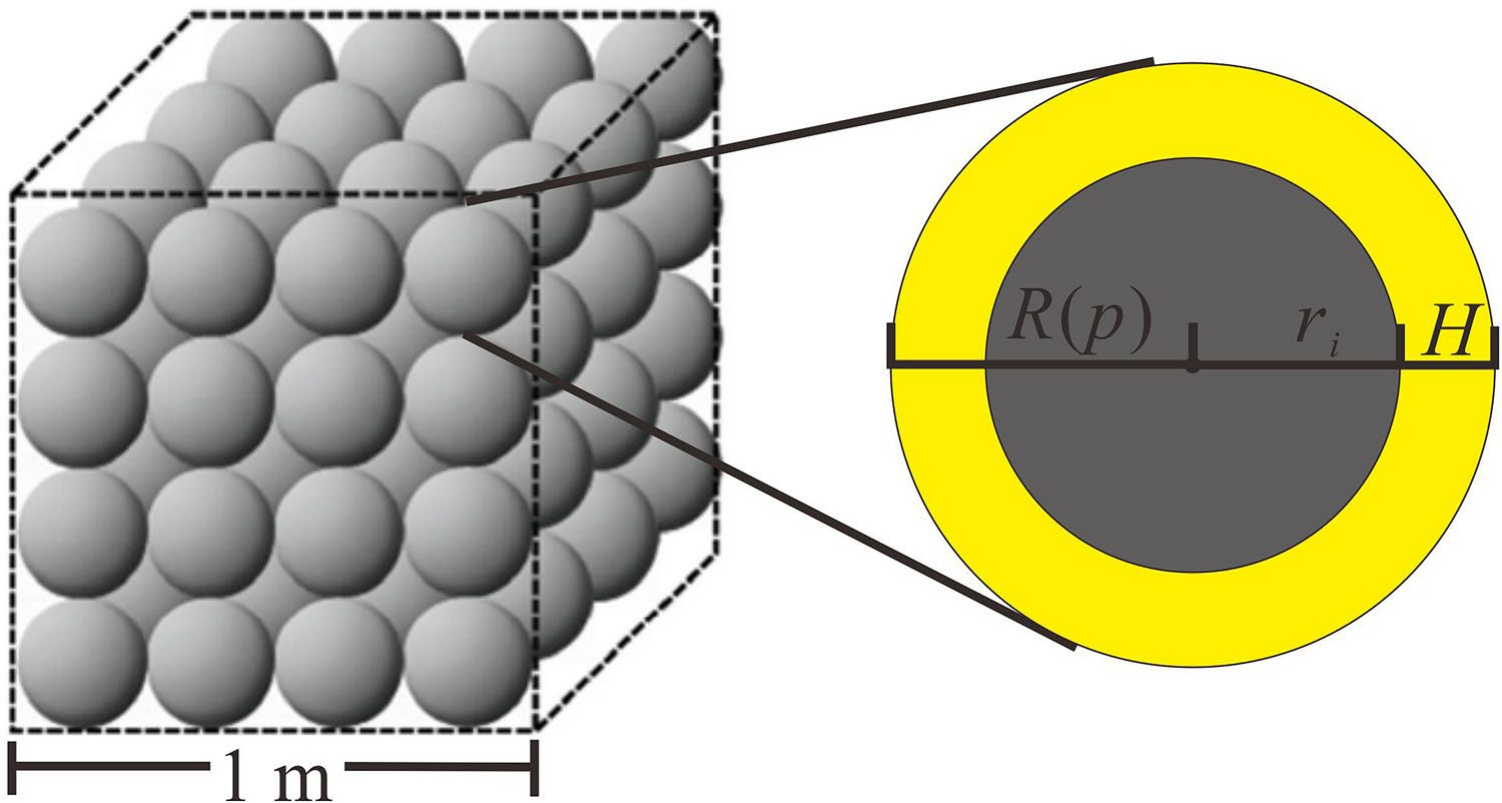
The equivalent matrix particle model of coal.

For matrix shrinkage induced permeability, Lai et al.^22^ proposed an equivalent matrix particle model, in which the matrix unit is regarded as a cube containing a defined number (n^3^) of matrix particles, and a volume ($V_{f}$) of 1 m^3^ (Fig. 4). Thus, the porosity variation can be described as follow^22^:

3 $$\Delta {{\varphi }} = \frac{{4\pi n^{3} }}{{3V_{f} }}\left( {R\left( {P_{cd} } \right)^{3} - R\left( {P_{g} } \right)^{3} } \right)$$

$R\left( P \right)$ is defined as the equivalent matrix particle radius, which is the sum of the matrix radius and the adsorption layer thickness as described in Eq. (4):

4 $$R\left( P \right) = H + r_{i} = \frac{{10^{ - 3} \rho V\left( {V_{L} P} \right)}}{{\rho VS_{v} \left( {P_{L} + P} \right)}} + r_{i}$$

where $H$ is the adsorption layer thickness, m; $r_{i}$ is the matrix particle radius, m; $\rho$ is the coal density, g/cm^3^; $V$ is the coal volume, m^3^; $V_{L}$ is the Langmuir volume, m^3^/t; $S_{v}$ is the specific surface area, m^2^/kg. The relationship between $S_{v}$ and $r_{i}$ is expressed as^23^:

5 $$r_{i} = \frac{{3 \times 10^{ - 3} }}{{S_{v} \rho }}$$

According to the classical P&M model^24^, the relationship between the porosity and permeability can be obtained as follow:

6 $$k\left( P \right) = k_{i} \left[ {\frac{\varphi \left( P \right)}{{\varphi i}}} \right]^{3}$$

By combining the calculation Eqs. (3, 4, 5) with Eq. (6), a general formula can be established for calculating dynamic permeability induced by the matrix shrinkage effect:

7 $$k_{shr} = k_{cd} \left[ {\frac{{\frac{{\pi S_{v}^{3} \rho^{3} }}{162}\left( {R\left( {P_{cd} } \right)^{3} - R\left( {P_{g} } \right)^{3} } \right) + \varphi_{i} }}{{\varphi_{i} }}{ }} \right]^{3}$$

where $k_{shr}$ is the permeability induced by the matrix shrinkage effect, mD; $k_{cd}$ is the permeability when the reservoir pressure is $P_{cd}$, mD.

The slippage effect is ignored as its effect on permeability is only one-tenth of the matrix shrinkage effect^25–27^. Therefore, permeability induced by the effective stress effect and the matrix shrinkage effect can be expressed as follow:

8 $$k = k_{i} + \Delta k_{eff} + \Delta k_{shr}$$

Substituting Eqs. (2, 7) into Eq. (8), dynamic permeability in the desorption area can be finally established:

9 $$k = k_{i} e^{{ - C_{f} \left( {\frac{{1 + v}}{{1 - v}}} \right)\left( {P_{i} - P_{g} } \right)}} + k_{i} e^{{ - C_{f} \left( {\frac{{1 + v}}{{1 - v}}} \right)\left( {P_{i} - P_{{cd}} } \right)}} \left[ {\frac{{\frac{{\pi S_{v} ^{3} \rho ^{3} }}{{162}}\left( {R\left( {P_{{cd}} } \right)^{3} - R\left( {P_{g} } \right)^{3} } \right) + \varphi _{i} }}{{\varphi _{i} }}{\text{~}}} \right]^{3} - k_{i} e^{{ - C_{f} \left( {\frac{{1 + v}}{{1 - v}}} \right)\left( {P_{i} - P_{{cd}} } \right)}}$$

**Figure 5:**
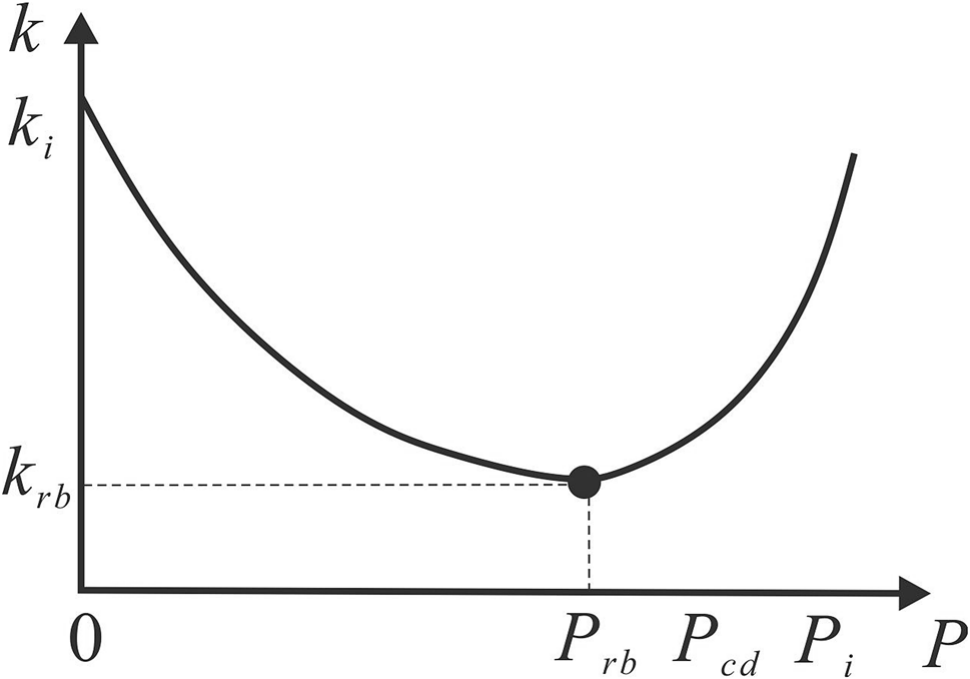
Dynamic change in coal reservoir permeability with continued production.

Previous studies have shown that the relationship between permeability and reservoir pressure is parabolic (Fig. 5). In the early stage, the damage effect is dominant and reservoir permeability continually decreases, while the recovery effect increases with gas desorption. When the permeability damage induced by effective stress is equal to the permeability recovery induced by matrix shrinkage, reservoir permeability decreases to a minimum level (${k}_{rb}$) and the corresponding reservoir pressure is the rebound pressure (${P}_{rb}$), which is provided by the solution to ${k}^{^{\prime}}$=0 as described in Eqs. (10) and (11)^25,27^.

10 $$k^{\prime } = C_{f} \left( {\frac{{1 + v}}{{1 - v}}} \right)k_{{cd}} e^{{ - C_{f} \left( {\frac{{1 + v}}{{1 - v}}} \right)(P_{{cd}} - P_{{rb}} )}} - \frac{{9\pi S_{v} ^{3} \rho ^{3} }}{{162}}k_{{cd}} R\left( {P_{{rb}} } \right)^{2} R\left( {P_{{rb}} } \right)^{\prime } \left[ {\frac{{\frac{{\pi S_{v} ^{3} \rho ^{3} }}{{162}}\left( {R\left( {P_{{cd}} } \right)^{3} - R\left( {P_{{rb}} } \right)^{3} } \right) + \varphi _{i} }}{{\varphi _{i} }}} \right]^{2} = 0$$

11 $$k_{{rb}} = k|_{{P = P_{{rb}} }}.$$

## Model construction

### Optimization of drainage strategy in the single-phase water flow stage

In the single-phase water flow stage, reservoir pressure is reduced by dewatering, and permeability decreases linearly with the pressure depletion. Thus, a reasonable drainage strategy needs to be established to cause the pressure propagation radius to expand sufficiently.

Water saturation is equal to the initial value as there is no gas desorbed in this stage, so water production comes from the increase in water volume, which is caused by the change in water compressibility, the occurrence of elastic expansion and the decrease in porosity during depressurization. The material balance formula in differential form is written as:

12 $$dW_{P} = \frac{{S_{{wi}} }}{{B_{w} }}\left[ {\varphi _{i} + \varphi _{i} C_{w} \left( {P_{i} - P_{w} } \right) - \varphi _{1} } \right]dV .$$

13 $$dV = hdA = 2\pi rhdr$$

According to the continuous succession of steady states concept, transient flow is considered as a succession of steady states^28–31^. Therefore, the pressure profile in the drainage area is expressed as follow^10^:

14 $$P_{w} = P_{cd} + \frac{{P_{i} - P_{cd} }}{{ln\left( {\frac{{r_{e} }}{{r_{w} e^{ - S} }}} \right)}}ln\left( {\frac{r}{{r_{w} e^{ - S} }}} \right)$$

By substituting Eqs. (1), (13), and (14) into Eq. (12), the integration of water production in the whole region can be obtained.

15 $$W_{P} = \frac{{2\pi h\varphi_{i} S_{wi} }}{{B_{w} }}\mathop \int \limits_{{r_{w} }}^{{r_{e} }} \left[ {\left( {C_{w} + C_{f} } \right)\left( {P_{i} - P_{w} } \right)r} \right]dr$$

According to Darcy's law, the water flow rate under the pressure difference is:

16 $$q_{w} = \frac{{542.87k_{1} h\left( {P_{i} - P_{cd} } \right)}}{{B_{w} \mu_{w} \ln \left( {\frac{{r_{e} }}{{r_{w} e^{ - S} }}} \right)}}$$

Then, the maximum BHFP drop rate in the stage can be calculated:

17 $$t_{1} = \frac{{W_{P} }}{{q_{w} }}$$

18 $$v_{1} = \frac{{P_{i} - P_{cd} }}{{t_{1} }}$$

where because artificial fractures play a major role in the flow of water in the single-phase water flow stage, $k_{1}$ is the permeability of fractures, mD. $r_{e}$ is well-controlled radius, m; $\mu_{w}$ is water viscosity, ${\text{mpa}} \cdot {\text{s}}$; $W_{P}$ is the cumulative water production in the single-phase water flow stage, m^3^; $B_{w}$ is the water formation volume coefficient, dimensionless, and approximately equal to 1; $C_{w}$ is the formation water compressibility coefficient, MPa^-1^;$S_{wi}$ is the initial water saturation, dimensionless;$A$ is the well-controlled area, m^2^; $h$ is coal reservoir thickness, m; ${\text{ S}}$ is the skin factor, dimensionless; $r_{w}$ is the wellbore radius, m; $v_{1}$ and $t_{1}$ refer to the maximum BHFP drop rate and the production time in the single-phase water flow stage, MPa/d and day, respectively.

It is worthy of note, water production refers to formation water rather than fracturing fluid for CBM wells with artificial fracturing. Additionally, BHFP can only reduce to below the critical desorption pressure when the pressure propagation radius reaches $r_{e}$ and formation water is fully produced. Otherwise, coal reservoirs are not only damaged by a stress-sensitive effect, but also blocked by the coupled relationship between gas and water, resulting in insufficient pressure propagation.

### Optimization of drainage strategy in the gas–water flow stage

Gas desorption is initiated when the BHFP drops below the critical desorption pressure, allowing CBM production to enter the gas–water flow stage. The aim of this stage is to expand $r_{cd}$ to $r_{e}$ and thus, it is crucial to assess whether the average reservoir pressure ($\overline{P}$) can reach $P_{rb}$ during pressure propagation. If this can be achieved, reservoir permeability increases with subsequent production and massive CBM desorption will contribute to the recovery of reservoir permeability, instead of damaging the coal reservoir. Otherwise, a rapid drop in BHFP or a rapid increase in gas production can cause a decrease in reservoir permeability and inhibit expansion of the desorption radius, resulting in inefficient depressurization and low gas production in CBM wells. By matching the drainage strategies of CBM wells with the dynamic geological conditions, a secure principle is proposed to reasonably guide CBM production and prevent coal reservoir damage. The optimal process is outlined in detail:

1. Calculate BHFP at the end of the gas–water flow stage ($P_{wf}$). The pressure profile in the desorption area is described by the pressure-squared approach (Eq. 19)^30,31^ and its average pressure is further described by Eq. (20). Based on these equations, it can be seen that the geological properties and reconstruction of coal reservoirs directly affect the ability of the average reservoir pressure to reach $P_{rb}$ during the decrease of BHFP. If the average reservoir pressure can reach $P_{rb}$, the BHFP value ($P_{wf}$) is expressed as $P_{rwb}$ (Fig. 6a). However, if the average reservoir pressure remains greater than $P_{rb}$ until the BHFP drops to the abandoned pressure ($P_{ab}$), $P_{wf}$ is equal to $P_{ab}$ (Fig. 6b).
  19 $$P_{g}^{2} = P_{wf}^{2} + \frac{{P_{cd}^{2} - P_{wf}^{2} }}{{ln\left( {\frac{{r_{e} }}{{r_{w} e^{{ - {\text{S}}}} }}} \right)}}ln\left( {\frac{r}{{r_{w} e^{{ - {\text{S}}}} }}} \right)$$
  20 $$\bar{P} = \frac{{\mathop \smallint \nolimits_{{r_{w} }}^{{r_{e} }} P_{g} dA}}{A} = \frac{{2\pi \mathop \smallint \nolimits_{{r_{w} }}^{{r_{e} }} \left( {P_{{wf}} ^{2} + \frac{{P_{{cd}} ^{2} - P_{{wf}} ^{2} }}{{ln\left( {\frac{{r_{e} }}{{r_{w} e^{{ - {\text{S}}}} }}} \right)}}ln\left( {\frac{r}{{r_{w} e^{{ - {\text{S}}}} }}} \right)} \right)^{{\frac{1}{2}}} rdr}}{{\pi \left( {r_{e} ^{2} - r_{w} ^{2} } \right)}}$$
  21 $$\left\{ {\begin{array}{*{20}l} {P_{wf} = P_{rbw} } \hfill & {\quad \left( {\overline{P} \le P_{rb} } \right)} \hfill \\ {P_{wf} = P_{ab} } \hfill & {\quad \left( {\overline{P} > P_{rb} } \right)} \hfill \\ \end{array} } \right.$$

**Figure 6:**
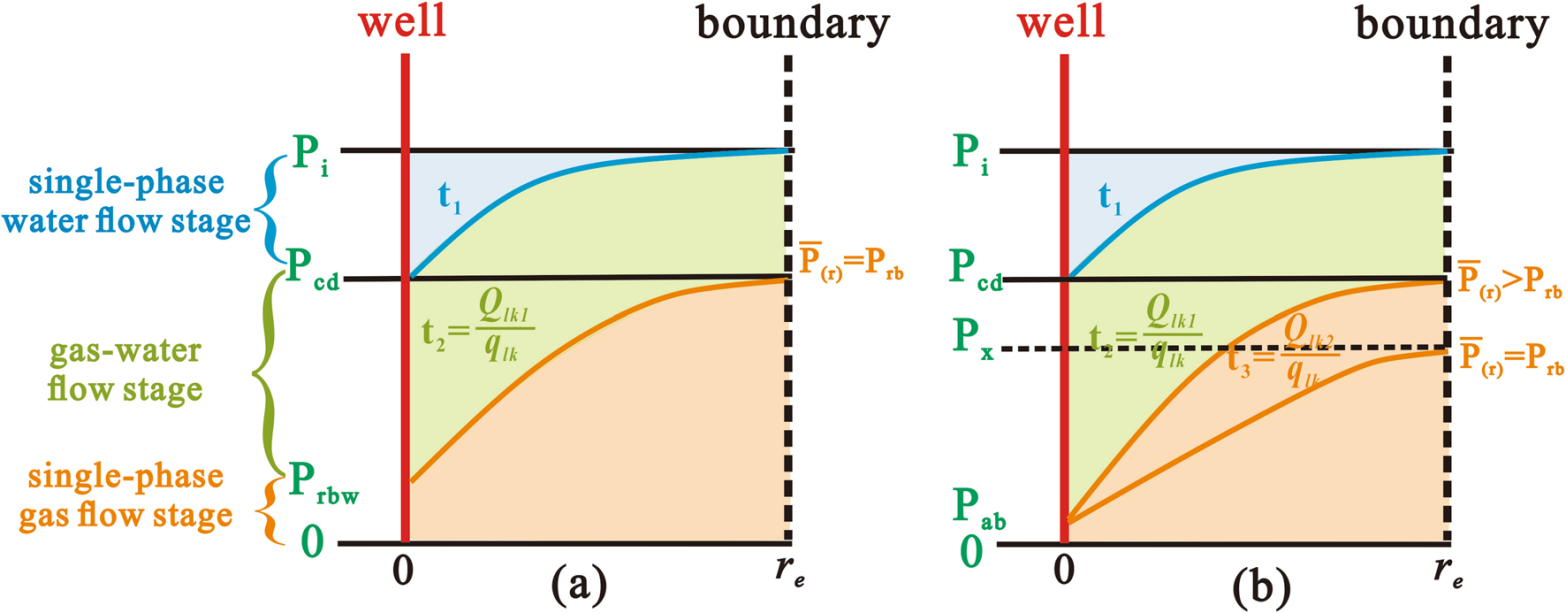
The optimal drainage strategy model for different coal reservoir conditions. (**a**) Average reservoir pressure can be reduced to rebound pressure in the gas–water flow stage; (**b**) Average reservoir pressure can be reduced to rebound pressure in the single-phase gas flow stage.
  where, $P_{wf}$ is BHFP, MPa; $P_{rbw}$ is BHFP corresponding to $\overline{P} = P_{rb}$, MPa;$P_{ab}$ is the abandoned pressure, MPa.
2. Calculate the cumulative gas production. The ground volume of accumulated gas production is equal to the ground volume of accumulated gas desorption plus the ground volume of initial free gas, with the remaining geological reserves of adsorbed gas in the fracture then subtracted^32^, the material balance formula in differential form is written as:
  22 $$dQ_{lk1} = \left[ {\frac{{\rho V_{L} P_{cd} }}{{P_{L} + P_{cd} }} - \frac{{\rho V_{L} P_{g} }}{{P_{L} + P_{g} }} + \frac{{\varphi_{i} \left( {1 - S_{wi} } \right)}}{{B_{gi} }} - \frac{{\varphi_{2} \left( {1 - S_{w} } \right)}}{{B_{g} }}} \right]dV$$
  By integration of gas desorption in the whole region, the cumulative production is given below:
  23 $$Q_{{lk1}} = 2\pi h\int_{{r_{w} }}^{{r_{e} }} r \left\lceil {\frac{{\rho V_{L} P_{{cd}} }}{{P_{L} + P_{{cd}} }} - \frac{{\rho V_{L} P_{g} }}{{P_{L} + P_{g} }} + \frac{{\varphi _{i} (1 - S_{wi} )}}{{B_{gi} }}- \frac{{\varphi _{i} (1 - S_{w} )}}{{B_{g} }}\left( {1 - C_{f} (P_{{cd}} - P_{g} ) + \varepsilon _{{\max }} \left( {\frac{{P_{{cd}} }}{{P_{L} + P_{{cd}} }} - \frac{{P_{g} }}{{P_{L} + P_{g} }}} \right)} \right)} \right\rceil dr$$
  24 $$B_{g} = 3.458 \times 10^{{ - 4}} Z\frac{{273 + T}}{{P_{{wf}} }}$$
  where ${\text{Q}}_{lk1}$ is cumulative gas production in the gas–water flow stage, m^3^; $B_{g}$ is the gas volume factor, dimensionless; Z is the deviation factor of gas and *T* is the reservoir temperature, which are assumed to be 1 and 23 °C respectively due to slight change during production.
3. Calculate the maximum daily gas production. In order to prevent a severe gas lock effect, the maximum daily gas production of the CBM well under the pressure difference was calculated according to Darcy's law^33^ (Eq. 25). The rebound permeability ($k_{rb}$) should be substituted into the equation according to the secure principle. It can be intuitively seen that the maximum daily gas production is proportional to permeability, critical desorption pressure and skin factor, while being inversely proportional to gas viscosity and the BHFP.
  25 $$q_{lk} = \frac{{542.87k_{rb} h\left( {P_{cd}^{2} - P_{wf}^{2} } \right)}}{{B_{g} P_{wf} \mu_{g} \ln \left( {\frac{{r_{e} }}{{r_{w} e^{ - S} }}} \right)}}$$
  where $q_{lk}$ is the maximum daily gas production, m^3^/d; and $\mu_{g}$ is the viscosity of water, ${\text{mpa}} \cdot {\text{s}}$.
4. Calculate the maximum BHFP drop rate. The ratio of cumulative gas production to maximum daily gas production indicates the shortest production time in the gas–water flow stage, allowing the corresponding maximum BHFP drop rate to be further calculated.
  26 $$t_{2} = \frac{{{\text{Q}}_{lk1} }}{{q_{lk} }}$$
  27 $$v_{2} = \frac{{\left( {P_{cd} - P_{wf} } \right)}}{{t_{2} }}$$
  where $t_{2}$ is the shortest production time in the gas–water flow stage, d; $v_{2}$ is the maximum pressure drop rate in the gas–water flow stage, MPa/d.

### Optimization of drainage strategy in the single-phase gas flow stage

When $r_{cd}$ reaches $r_{e}$, CBM production enters the single-phase gas flow stage. Due to the formation of inter-well pressure interference and massive CBM desorption, gas is dominant in pores, with almost no water production in CBM wells. In addition, because BHFP is reduced to a low level, it only requires minor adjustment. Thus, the key is to analyze whether artificial control over daily gas production is necessary.

As discussed previously, if the average reservoir pressure is greater than $P_{rb}$ at the end of the gas–water flow stage, massive CBM desorption will block pores because of the decrease in permeability. Therefore, casing pressure should be controlled to limit gas production until the average reservoir pressure reaches $P_{rb}$. According to Eqs. (28, 29), boundary pressure ($P_{x}$) is calculated based on the influence of inter-well pressure interference.

28 $$P_{g}^{2} = P_{wf}^{2} + \frac{{P_{x}^{2} - P_{wf}^{2} }}{{ln\left( {\frac{{r_{e} }}{{r_{w} e^{{ - {\text{S}}}} }}} \right)}}ln\left( {\frac{r}{{r_{w} e^{{ - {\text{S}}}} }}} \right)$$

29 $$\overline{P} = \frac{{2\pi \mathop \smallint \nolimits_{{r_{w} }}^{{r_{e} }} \left( {P_{wf}^{2} + \frac{{P_{x}^{2} - P_{ab}^{2} }}{{ln\left( {\frac{{r_{e} }}{{r_{w} e^{{ - {\text{S}}}} }}} \right)}}ln\left( {\frac{r}{{r_{w} e^{{ - {\text{S}}}} }}} \right)} \right)^{\frac{1}{2}} rdr}}{{\pi \left( {r_{e}^{2} - r_{w}^{2} } \right)}} = P_{rb}$$

The cumulative gas production and time required for this process can be obtained (Fig. 6b):

30 $$Q_{lk2} = \pi h\rho r_{e}^{2} \frac{{V_{L} P_{cd} }}{{P_{L} + P_{cd} }} - 2\pi h\mathop \smallint \limits_{{r_{w} }}^{{r_{e} }} \left( {r\frac{{\rho V_{L} P_{g2} }}{{P_{L} + P_{g2} }} + \frac{{\varphi_{2} \left( {1 - S_{w} } \right)}}{{B_{g} }}} \right)dr - Q_{lk1}$$

31 $$t_{3} = \frac{{{\text{Q}}_{lk2} }}{{q_{lk} }}$$

where $P_{x}$ is the boundary pressure when the average reservoir pressure reaches $P_{rb}$, MPa; $Q_{lk2}$ is cumulative gas production of CBM well in the single-phase gas flow stage according to $\overline{P} = P_{rb}$, m^3^; $t_{3}$ is the corresponding time, d.

### Calculation procedures

**Figure 7:**
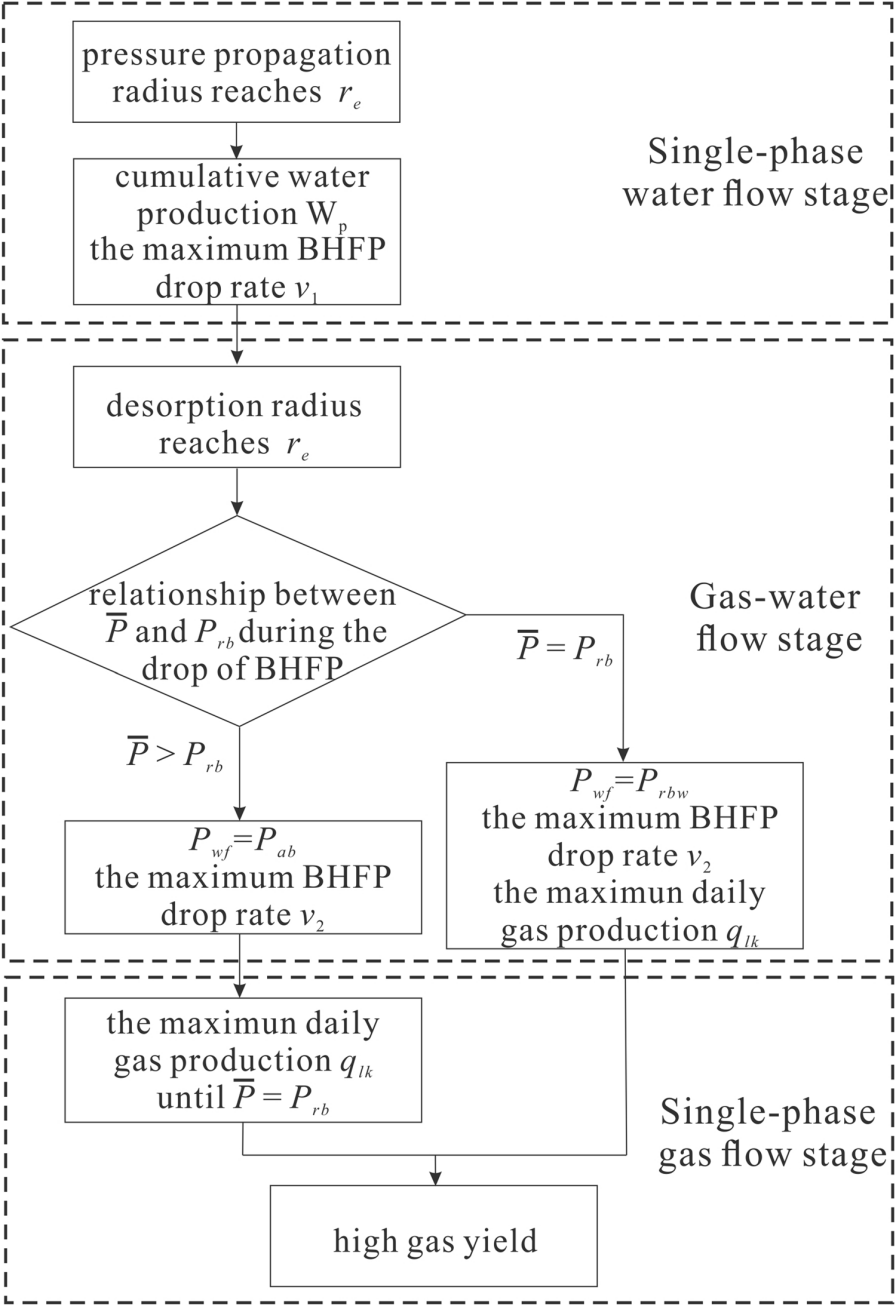
Analysis flow chart for optimization of CBM well drainage strategies.

Based on the drainage strategy optimization process for each stage, a mathematical model was established to guide CBM development (Fig. 7). Firstly, the BHFP drop rate and cumulative water production in the single-phase water flow stage were calculated according to the pressure propagation formula. Secondly, it was assessed whether the average reservoir pressure can reduce to the rebound pressure at the end of the gas–water flow stage, allowing the corresponding BHFP to be calculated and the maximum daily gas production and maximum BHFP drop rate to be obtained according to the safety principle. Finally, the casing pressure can be controlled to limit gas production in the single-phase gas flow stage, until average reservoir pressure drops below the rebound pressure.

## Results and discussion

### Selection of target wells

**Figure 8:**
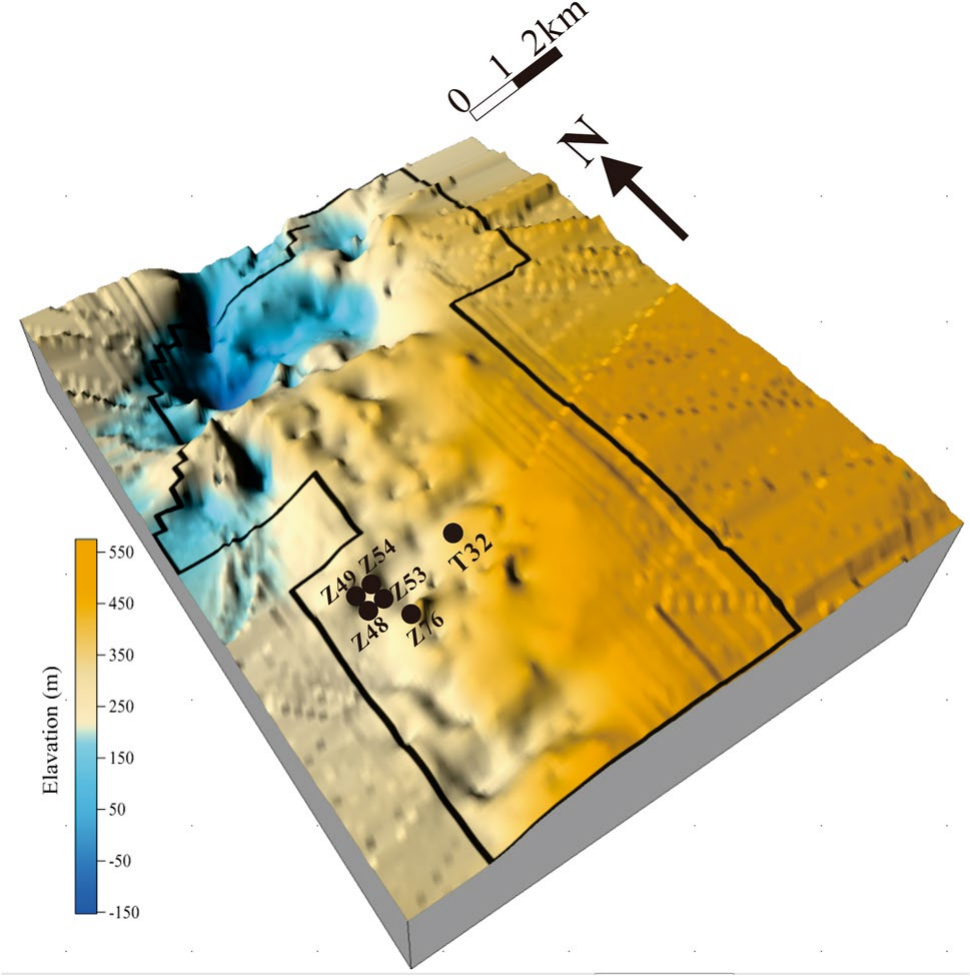
Elevation of coal seam No.3 and location of the target CBM wells in the area.

The regional geology of the Shizhuangnan Block in the Qinshui Basin (China) has been described in detail in previous articles^34–36^. In this study, six target wells were selected to assess the proposed method for quantitative optimization of drainage strategies (Fig. 8). Among these wells, Z48, Z49, Z53 and Z54 were adjacent wells, with a distance between wells of 300 m. According to logging and experimental data, these adjacent wells exhibited similar geological structures, reservoir conditions and reconstruction degrees, and were not affected by natural faults or collapse columns. Moreover, the coal reservoir in the area is characterized as a typical high-rank anthracite reservoir, with low reservoir pressure and permeability and a high gas content. In contrast, the burial depth of well Z76 was relatively shallow, with this well being affected by natural fractures, leading to serious methane dissipation and high initial permeability. The original coal reservoir permeabilities of all test wells were generally less than 1 mD^37^, so that they had been hydrofractured. Some basic geological parameters were assessed experimentally via well tests, with data corrected by historical matching of numerical simulation. The Poisson’s ratio ($v$) was 0.3, the specific surface area (${\text{S}}_{{\text{v}}}$) was 1295 m^2^/kg, the cleat-volume compressibility ($C_{f}$) was 0.18 MPa^-1^, the formation water compressibility coefficient ($C_{w}$) was 0.00045 MPa^-1^, the maximum volumetric strain ($\varepsilon_{max}$) was 0.0325, the well-controlled radius ($r_{e}$) was 150 m, the wellbore radius ($r_{w}$) was 0.1 m, water viscosity ($\mu_{w}$) was 0.856 ${\text{mpa}} \cdot {\text{s}}$, gas viscosity ($\mu_{g}$) was 0.01134 ${\text{mpa}} \cdot {\text{s}}$, the initial water saturation ($S_{wi}$) was 0.95, and the irreducible water saturation was 0.6. Other specific parameters for each well are shown in Table 1.

**Table 1:** Geological parameters of the target wells.

Parameters	T32	Z76	Z48/Z49/Z53/Z54	Parameters	T32	Z76	Z48/Z49/Z53/Z54
Burial depth (m)	769	589	650	$P_{i}$(MPa)	3.3	2.9	4
*h* (m)	6	5.2	6	$P_{cd}$(MPa)	2.1	1.4	2.3
$k_{i}$(mD)	0.83	1.17	0.23	$P_{L}$(MPa)	1.5	2.5	1.7
$k_{1}$(mD)	4	5.3	4	$\varphi_{i}$	0.02	0.035	0.025
$V_{L}$(m^3^/t)	36	35	36	S	− 2.3	− 1	− 3
$\rho$(g/cm^3^)	1.5	1.2	1.4				

**Figure 9:**
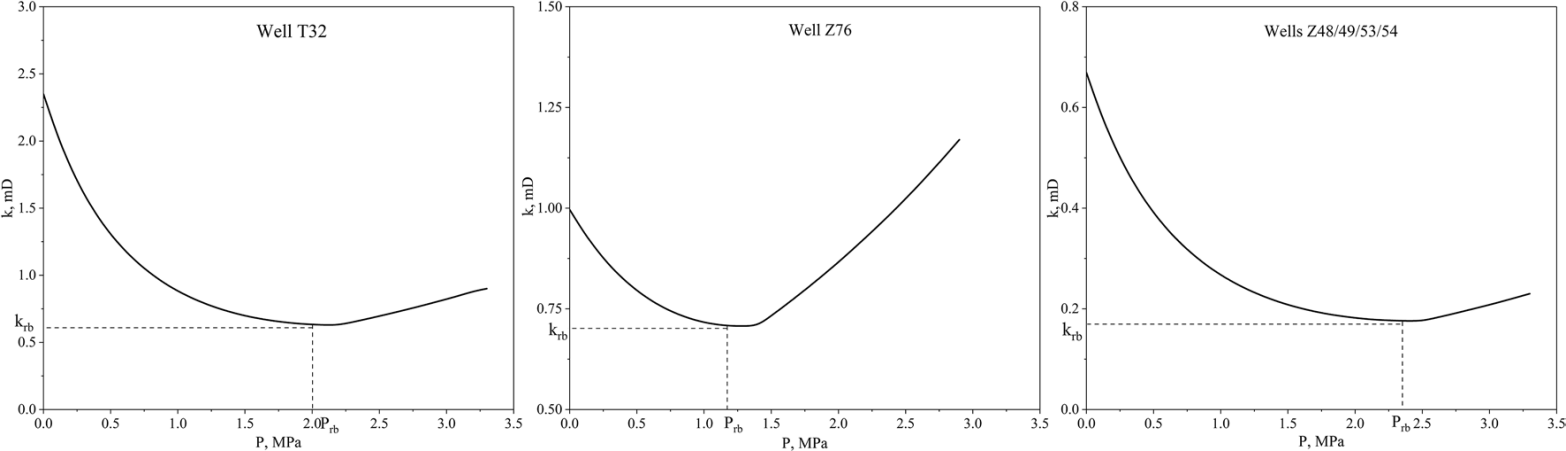
Dynamic permeability characteristics of the six target wells.

Additionally, because the geological parameters of coal reservoirs in the whole area exhibit obvious differences, the dynamic characteristics of permeability are unique. Therefore, the dynamic permeability of each well is described by Eqs. (2) and (9), with rebound points calculated by Eqs. (10) and (11) (Fig. 9). Results showed that the permeability of well Z76 was seriously damaged and did not easily recover to its initial level because of the low ratio of critical desorption pressure to initial reservoir pressure. However, the permeability of the other five wells increased significantly with massive gas desorption. The production strategies for these wells were further analyzed according to their different geological characteristics.

### Method validation

**Figure 10:**
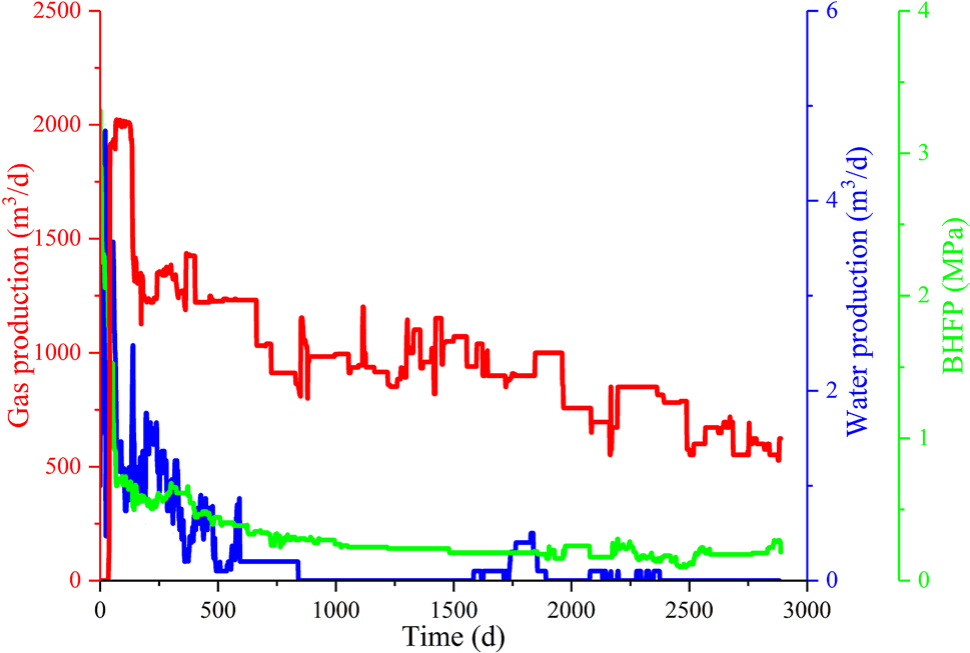
Production characteristics of well T32.

Well T32 was employed as an example well for validation of the proposed method. The production characteristics of well T32 showed that the BHFP decreased rapidly in the early stage and remained stable thereafter. Correspondingly, gas production in the early stage rapidly increased to a maximum value of 2500 m^3^/d and then continually declined after 140 days (Fig. 10). Therefore, it may be speculated that unreasonable production strategies lead to low CBM well gas yields.

A numerical simulation of well T32 was constructed using Comet3, which is widely applied and regarded as reliable reservoir simulation software in the CBM field. In the numerical simulation, (1) the actual BHFP was used to fit the actual CBM production, with geological parameters corrected by history matching; (2) production strategies were optimized by substituting the corrected parameters into the proposed model; (3) ideal CBM production rates were predicted by combining the optimized production strategies; (4) the practical applicability of the model was finally verified by comparing the actual and predicted gas production rates.

**Figure 11:**
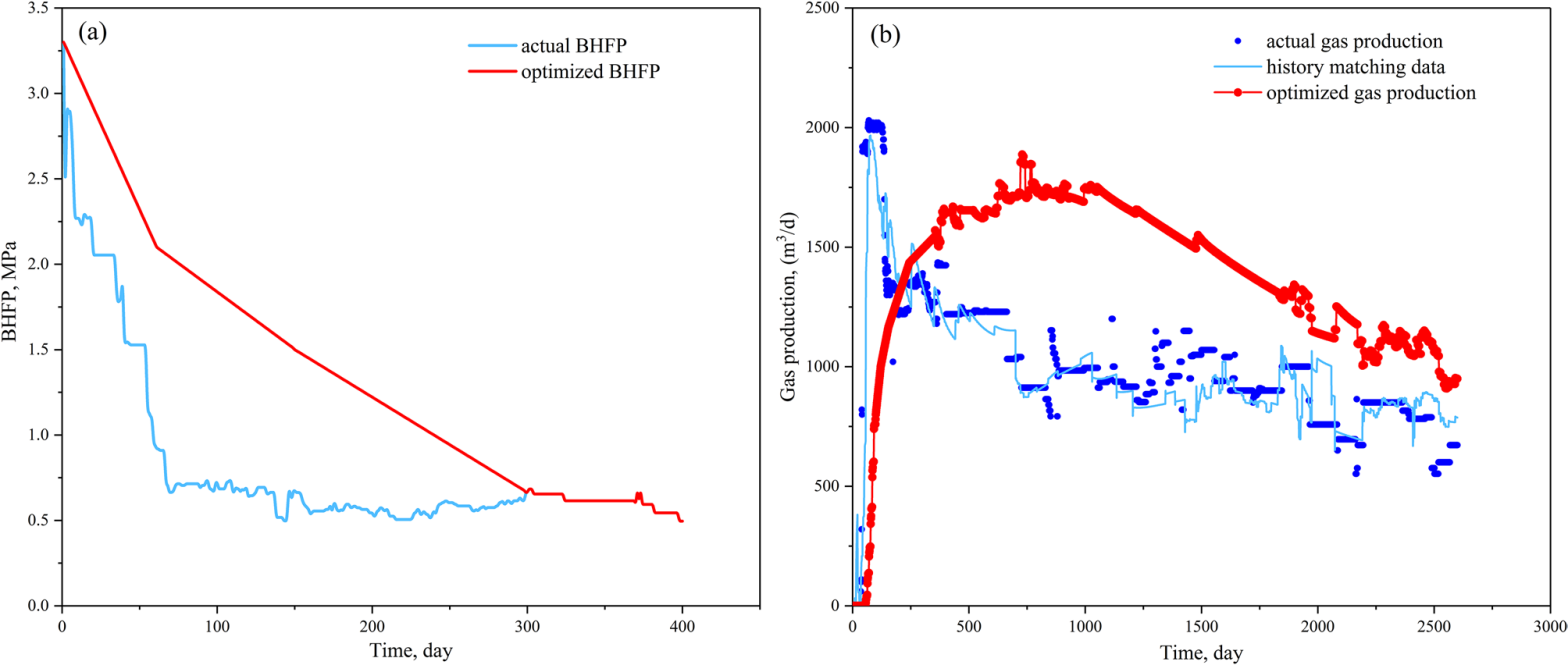
Numerical simulation results for well T32: (**a**) comparison of actual and optimized BHFP values; (**b**) comparison between actual gas production rates and optimized gas production rates.

**Figure 12:**
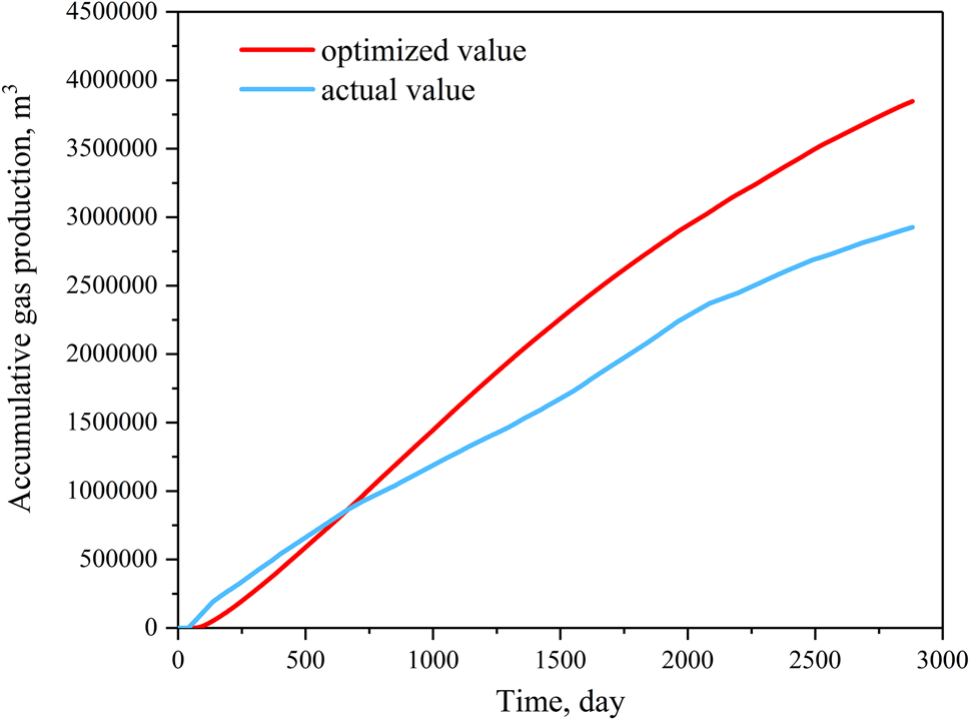
Comparison between actual and optimized cumulative gas production yield of well T32.

Optimized results and productivity prediction are shown in Table 2 and Fig. 11, respectively (the simulated production strategy values were slightly smaller than the calculated results due to the secure principle). Results showed that the decrease rate of the optimized BHFP was much slower than the actual values in the single phase water flow stage and gas–water flow stage. As a result, when the optimized BHFP reduced to 0.6 MPa, the desorption radius extended to the boundary and the average reservoir pressure reached the rebound pressure, causing gas production in the CBM well to increase, with high yields maintained throughout the single phase gas flow stage. In addition, although the actual cumulative gas production is greater in the early stage, optimized cumulative gas production exceeds the actual value after 700 days of production (Fig. 12). In general, the proposed model provides a time-saving and practical method to optimize CBM well production strategies, with reliability of the model successfully verified based on the comparison of simulated gas production to actual gas production in a CBM well.

**Table 2:** Calculated results of quantitative optimization of drainage strategies for well T32.

Parameters	Calculation results	Parameters	Actual values	Calculation results
$P_{rb}$(MPa)	2	$W_{P}$(m^3^)	80	145
$P_{rbw}$(MPa)	0.6	$v_{1}$(KPa/d)	60.0	16.4
$k_{rb}$(mD)	0.6	$t_{1}$(d)	20	61
$q_{w}$(m^3^/d)	2.4	$q_{lk}$(m^3^/d)	1859	1341
$Q_{lk1}$(m^3^)	241,729	$v_{2}$(KPa/d)	30	8.3
		$t_{2}$(d)	50	180

### Example of reasonable production strategy

According to the proposed mathematical model, the drainage strategies of well Z76 were quantitatively optimized (Table 3). The maximum BHFP drop rate was 26.8 kPa/d for 57 days in the single-phase water flow stage, reducing to 4.1 kPa/d for 292 days in the gas–water flow stage. It is worth noting that when the BHFP drops to the abandoned pressure, the average reservoir pressure is greater than $P_{rb}$, which indicates that artificial control of the casing pressure is necessary to limit daily gas production until the average reservoir pressure reaches $P_{rb}$. Therefore, the calculated maximum daily gas production was 510 m^3^/d for 502 days in the single-phase gas flow stage based on the actual reservoir conditions.

**Table 3:** Calculated results of quantitative optimization of drainage strategies for well Z76.

Parameters	Calculation results	Parameters	Calculation results	Actual values
$\overline{P}$(Mpa)	1.34	$W_{P}$(m^3^)	242	231
$P_{rb}$(Mpa)	1.24	$v_{1}$(KPa/d)	26.8	18.6
$P_{x}$(Mpa)	1.3	$t_{1}$(d)	57	80
$k_{rb}$(Md)	0.7	$q_{lk}$(m^3^/d)	510	540
$q_{w}$(m^3^/d)	4.2	$v_{2}$(KPa/d)	4.1	2.9
$Q_{lk1}$(m^3^)	148,950	$t_{2}$(d)	292	343
$Q_{lk2}$(m^3^)	256,060	$t_{3}$(d)	502	497

**Figure 13:**
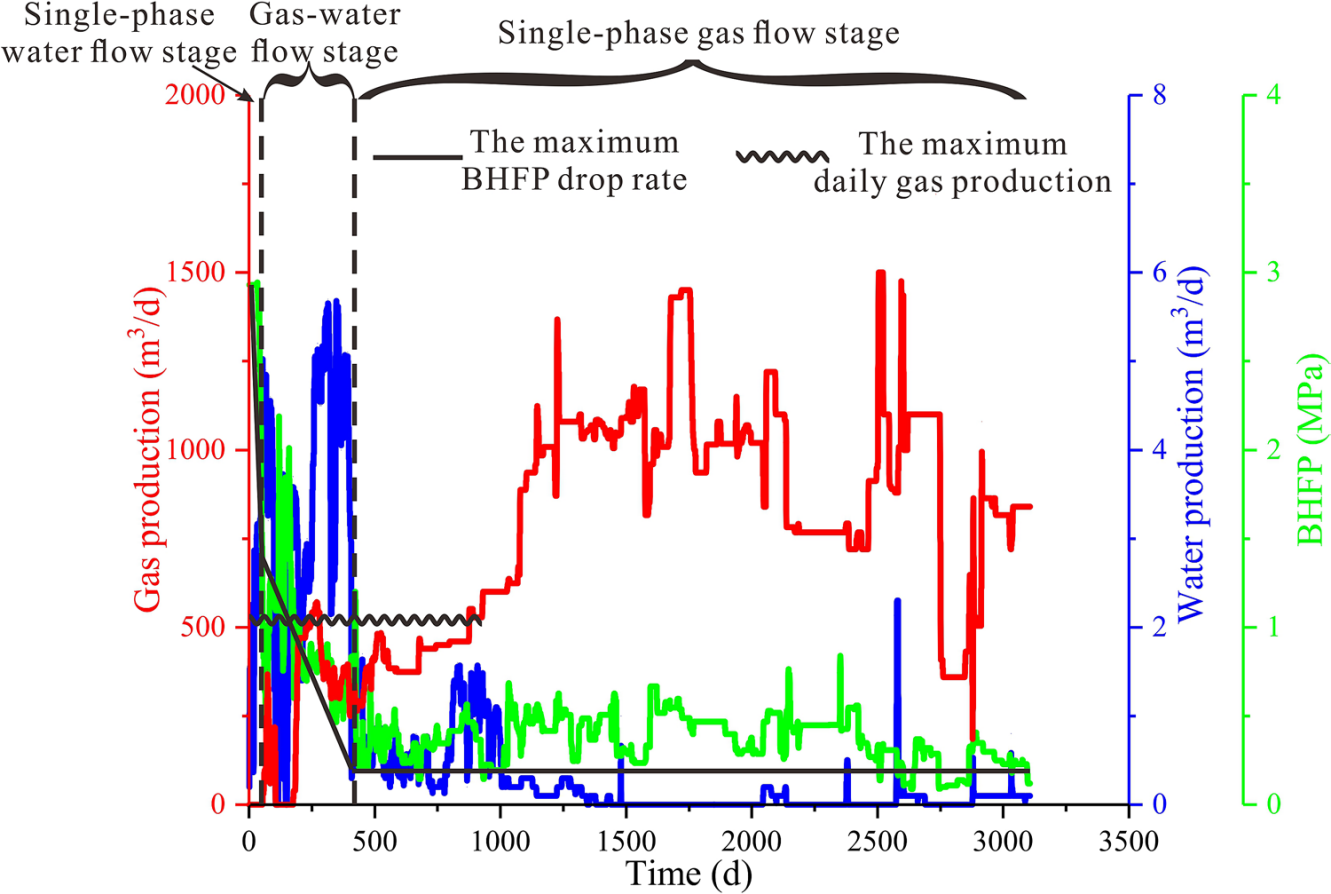
Production characteristics and quantitative optimization of the drainage strategy for the assessed target well Z76.

The actual drainage strategy results were similar to the calculation results: the actual BHFP drop rate was slightly less than the maximum BHFP drop rate, while the cumulative water production and daily gas production were generally consistent with the optimization results. Therefore, full pressure propagation and depressurization were conducive to high and stable gas production rates in the assessed well in the later stage of production, even if the gas content was low (12.56 m^3^/t). In addition, CBM wells primarily produce water in the early stage, with virtually no water production occurring in the single-phase gas flow stage (Fig. 13).

### Examples of unreasonable production strategies

For the four adjacent wells, the calculation results differed significantly from those of well Z76 (Table 4). In the single-phase water flow stage, the cumulative water production of a single well was 177 m^3^, and the maximum BHFP drop rate was 30.3 kPa/d for 62 days. The duration of the gas–water flow stage was 577 days, while the maximum BHFP drop rate and the maximum daily gas production rate were 3.20 kPa/d and 580 m^3^/d, respectively. Additionally, when the BHFP dropped to 0.5 MPa, the desorption radius reached $r_{e}$ and the average reservoir pressure was equal to $P_{rb}$, indicating that it was not necessary to adjust the BHFP substantially or limit the daily gas production of CBM wells in the later stage.

**Table 4:** Quantitative optimization of the drainage strategies for four adjacent wells.

Parameters	Calculation results	Parameters	Calculation results	Actual values			
				Z49	Z48	Z54	Z53
$P_{rb}$(MPa)	2.35	$W_{P}$(m^3^)	177	165	158	69	54
$P_{rbw}$(MPa)	0.5	$v_{1}$(KPa/d)	30.3	17	28.3	31.5	60.7
$k_{rb}$(mD)	0.15	$t_{1}$(d)	62	83	60	54	28
$q_{w}$(m^3^/d)	2.8	$q_{lk}$(m^3^/d)	580	570	530	2650	510
$Q_{lk1}$(m^3^)	334,241	$v_{2}$(KPa/d)	3.2	8.47	2.35	2.36	13.6
		$t_{2}$(d)	577	177	640	636	110

**Figure 14:**
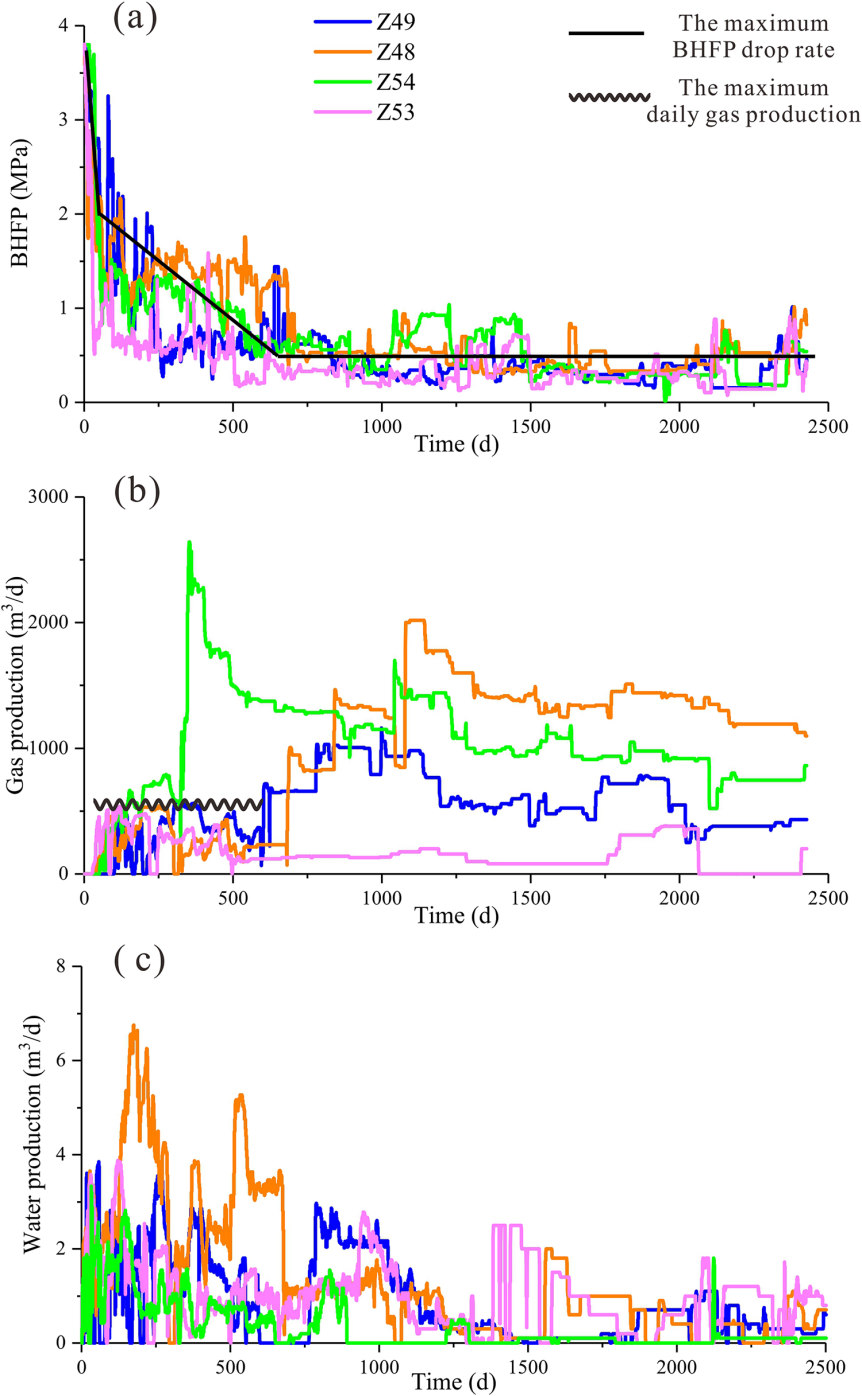
Quantitative optimization of drainage strategies for adjacent wells. (**a**) Comparison between the BHFP and optimization results; (**b**) comparison of daily gas production; (**c**) comparison of daily water production.

The calculated results were compared with the actual drainage strategies for the four adjacent wells (Fig. 14a). In the single-phase water flow stage, the actual BHFP drop rate of well Z53 was much larger than the calculated maximum value, while the values for all other wells’ were reasonably similar to the calculated values. Cumulative water production by well Z53 and well Z54 were far less than the calculated results, indicating that the pressure propagation radius was far away from the well-controlled boundary. In the gas–water flow stage, the actual drainage strategies for the four wells were significantly different: the BHFP drop rates of wells Z49 and Z53 were much larger than the optimized values, while that of well Z48 was slower. The daily gas productions of wells Z48, Z49, Z53 were less than the maximum daily gas production. However, for well Z54, although the BHFP drop rate was similar to the calculated results, the daily gas production was much greater than the calculated maximum (Fig. 14b).

Different drainage strategies resulted in obvious production differences between the target wells (Fig. 14b,c). Water production in well Z48 was sufficient, with the daily gas production consistently below 500 m^3^/t in the early stage, gradually increasing and reaching high yield in the single-phase gas flow stage. Average gas production rates of wells Z49 and Z53 were generally low during the whole production process, at about 510 m^3^/d and 161 m^3^/d, respectively, with intermittent water production. For well Z54, daily gas production increased rapidly and reached a peak gas production rate of 2500 m^3^/d about 350 days into the production process. Following this, gas production reduced rapidly and maintained a low yield. Moreover, well Z54 had minimal cumulative water production, with negligible water production in the later stage. Therefore, the effect of drainage strategies on the dynamic characteristics of the pressure drop funnel need to be further analyzed in detail.

### Characterization of pressure drop funnels

**Figure 15:**
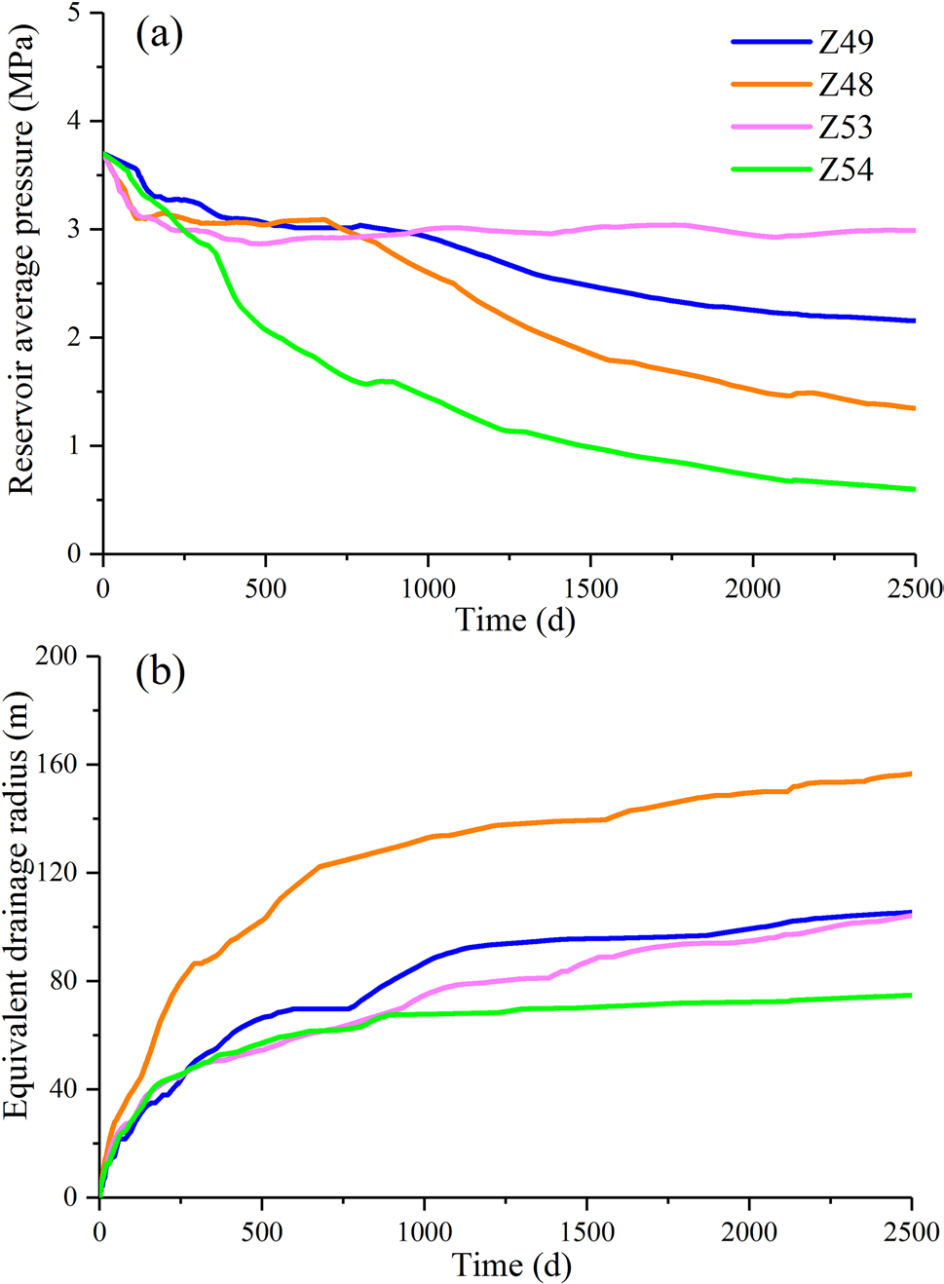
Dynamic characteristics of the coal reservoir of adjacent wells during CBM production. (**a**) Reservoir average pressure; (**b**) equivalent drainage radius.

A reservoir pressure prediction model based on the material balance equation was used to study the dynamic characteristics of reservoir pressure of the target wells during the CBM production process^38^. This model makes full use of the actual geological parameters and production data. Results showed that the pressure drop funnel of well Z48 propagated to the well-controlled boundary, indicating that inter-well interference occurred and depressurization was sufficient. However, the other assessed wells had not yet formed pressure interference. Notably, although the reservoir pressure of well Z54 exhibited an obvious drop, the small pressure propagation radius resulted in less CBM resources in the area, which was the most major cause of the rapid decrease in gas production in the later stage (Fig. 15a,b).

**Figure 16:**
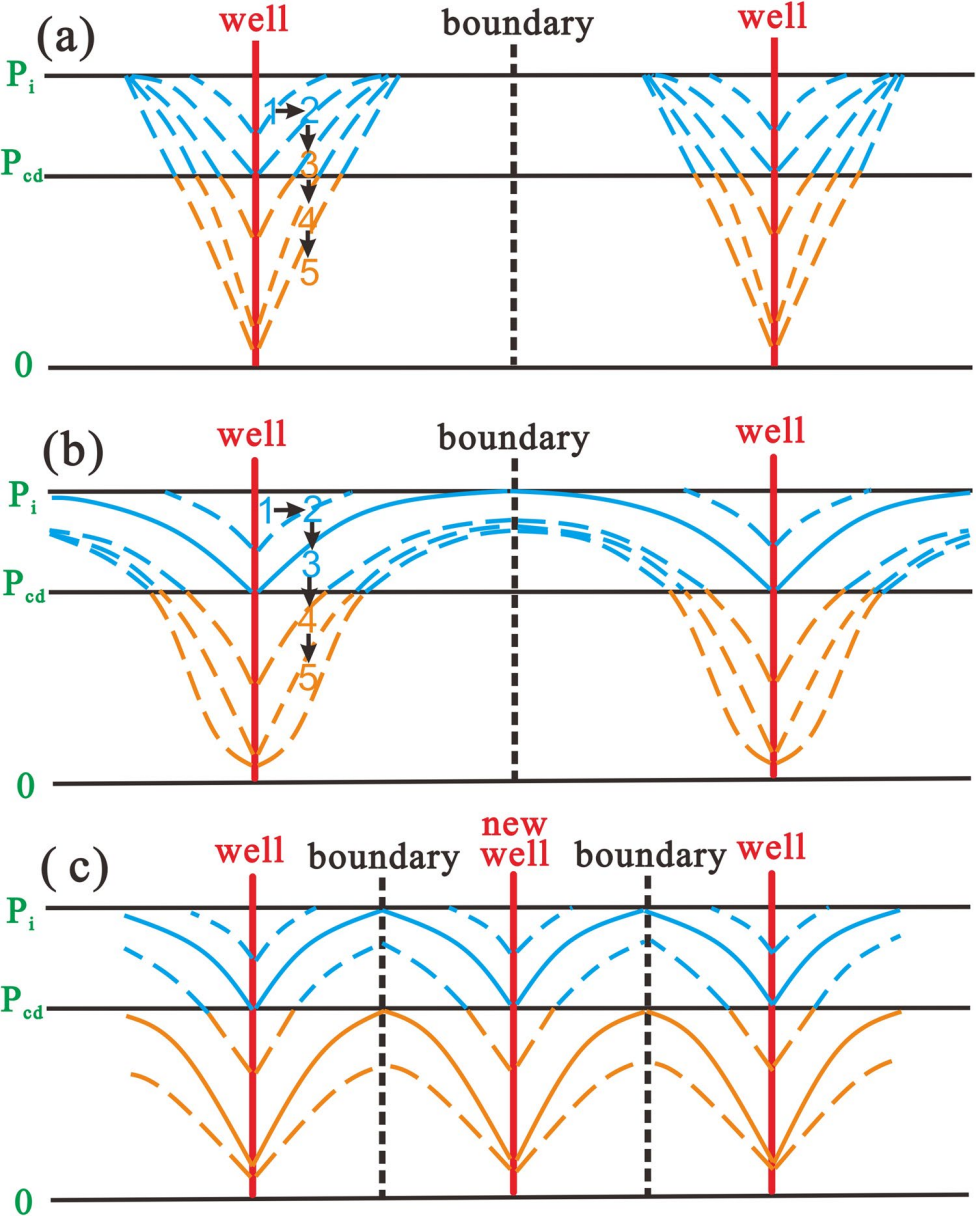
Characteristics of the pressure drop funnel in different situations. (**a**) Unreasonable drainage strategy in the single-phase water flow stage; (**b**) unreasonable drainage strategy in the gas–water flow stage; (**c**) infill well pattern.

In short, different drainage strategies lead to significant differences in pressure propagation and reservoir depressurization, as the pressure drop funnel characteristics differ. If the actual BHFP drops quicker than the maximum drop rate, water production will be far below the reasonable cumulative value, or gas production rates will be greater than the maximum production rate. Therefore, the coal reservoir is not only affected by stress-sensitive effects, but also by the water/gas lock effect. These effects can cause irreversible damage to the coal reservoir, inhibiting pressure propagation and ultimately leading to low gas production rates and intermittent water production in CBM wells (Fig. 16 a,b). However, if the BHFP drop rate and daily gas production rate is less than the reasonable value, the pressure drop funnel can fully expand, and the CBM well can achieve high gas yields. The economic benefit of the production well is reduced if the BHFP drops too slowly.

In order to overcome the adverse effects caused by unreasonable drainage strategies, engineering measures are crucial, such as refracturing and infill well drilling. The purpose of refracturing is to repair irreversible damage to the coal reservoir, gradually expanding the pressure drop funnel during later production stages. However, the purpose of infill well drilling is to shorten the well-controlled boundaries by drilling new wells (Fig. 16c). Both of these methods are ultimately conducive to multi-well pressure interference and efficient depressurization.

## Conclusions

1. The production stages in CBM wells were characterized based on dynamic change in the pressure drop funnel. In the single-phase water flow and gas–water flow stages, the pressure propagation radius and gas desorption radius should reach the well-controlled boundary, respectively. In the single-phase gas flow stage, inter-well pressure interference occurs.
2. The optimal drainage strategy model for CBM wells in different drainage stages was established based on seepage theory and the dynamic behavior of permeability, allowing the maximum BHFP drop rate, cumulative water production, and maximum daily gas production to be obtained in different production stages. The model was verified by numerical simulation. Based on the results of test wells case verification, the optimized production strategies should be adopted in the CBM production process to ensure production wells produce efficiently.
3. Characteristics of pressure drop funnels with unreasonable drainage strategies, such as a rapid decrease in BHFP and unlimited gas production, were analyzed. Results confirmed that the coal reservoir could be irreversibly damaged and expansion of the pressure drop funnel should be limited in these situations. Most of the pressure loss was found to occur in the two-phase region, with minimal formation of inter-well pressure interference.
